# Smoking in pregnancy, adolescent mental health and cognitive performance in young adult offspring: results from a matched sample within a Finnish cohort

**DOI:** 10.1186/s12888-016-1142-9

**Published:** 2016-12-01

**Authors:** Hugh Ramsay, Jennifer H. Barnett, Graham K. Murray, Pirjo Mäki, Tuula Hurtig, Tanja Nordström, Jouko Miettunen, Vesa Kiviniemi, Solja Niemelä, Zdenka Pausova, Tomas Paus, Juha Veijola

**Affiliations:** 1 Department of Psychiatry, Institute of Clinical Medicine, University of Oulu and Oulu University Hospital, Oulu, Finland; 2 St. Michael’s House, Dublin, Ireland; 3 Department of Psychiatry, University of Cambridge, Cambridge, UK; 4 Cambridge Cognition Ltd, Cambridge, UK; 5 Department of Child Psychiatry, Institute of Clinical Medicine, University of Oulu and Oulu University Hospital, Oulu, Finland; 6 Department of Psychiatry, Länsi-Pohja Healthcare District, Kemi, Finland; 7 Department of Psychiatry, the Middle Ostrobothnia Central Hospital, Kiuru, Finland; 8 Mental Health Services, Joint Municipal Authority of Wellbeing in Raahe District, Raahe, Finland; 9 Mental Health Services, Basic Health Care District of Kallio, Helsinki, Finland; 10 Visala Hospital, the Northern Ostrobothnia Hospital District, Oulu, Finland; 11 Center for Life Course Health Research, University of Oulu, Oulu, Finland; 12 Department of Radiology, Research Unit of Medical Imaging, Physics and Technology, University of Oulu, Oulu, Finland; 13 Department of Psychiatry, Lapland Hospital District, Rovaniemi, Finland; 14 The Hospital for Sick Children, University of Toronto, Toronto, ON Canada; 15 Rotman Research Institute, Baycrest, Toronto, ON Canada; 16 Departments of Psychology and Psychiatry, University of Toronto, Toronto, ON Canada; 17 Child Mind Institute, New York, NY USA

**Keywords:** Prenatal smoking, Cognition, Psychotic-like experiences, Inattention and hyperactivity

## Abstract

**Background:**

The association between prenatal exposure to maternal cigarette smoking (PEMCS) and adult cognition is debated, including if there are differences according to sex. We aimed to determine if there are associations between PEMCS and cognition in early adulthood in men and women and examine if observed associations were mediated by adolescent mental health factors that are associated with cognition, namely psychotic-like experiences (PLEs), inattention and hyperactivity, and other externalizing behaviors.

**Methods:**

Participants were 471 individuals drawn from the general population-based Northern Finland 1986 Birth Cohort (NFBC 1986) followed up from pregnancy and birth to early adulthood; individuals with PEMCS were matched with those without PEMCS by socioeconomic and demographic factors. Cognitive performance in adulthood was assessed with a range of tests and their association with PEMCS was measured by sex using hierarchical linear regression, unadjusted and then controlling for potential confounders, mediators and moderators, including adolescent mental health factors.

**Results:**

There were no associations between PEMCS and cognitive scores in females. In males, there were associations with vocabulary (beta = -0.444, 95% CI: -0.783, -0.104) and matrix reasoning (beta = -0.379, 95% CI: -0.711, -0.047).

**Conclusions:**

While associations between PEMCS and cognition were limited, observed findings with measures of general intelligence in males contribute to suggestions of differences in response to PEMCS by sex. Furthermore, observed associations may be partly mediated by earlier inattention and hyperactivity. Findings add support to efforts aimed to eliminate smoking in pregnancy.

## Background

Deficits in cognitive function are associated with a variety of adverse health and social outcomes [1]. While many determinants of cognitive function are not remediable through public health interventions, prenatal exposure to maternal cigarette smoking (PEMCS) is common and potentially preventable. Public health campaigns target PEMCS in order to prevent adverse outcomes in the offspring, including low birth weight and preterm birth [2]. PEMCS has been associated with medical problems [3] and with psychiatric and behavior problems in adolescence [4–6] and in adulthood [7–9].

In addition to these problems, a significant body of literature suggests that PEMCS may be harmful to cognitive functioning and development [10–17]. However, not all research supports this perspective [18–22] and a literature review of studies from 1972 to 1992 suggested that study limitations, such as poor control for covariates, prevented firm conclusions from being made [23]. More recent studies have addressed some of these limitations and in their systematic review of studies from 2000 to 2011, Clifford, Lang and Chen suggested more recent evidence supports a relationship, though one that is not straightforward [24], with the association most consistent for measures of academic achievement and intellectual ability. For example, in the Northern Finland 1966 Birth Cohort, PEMCS was strongly associated with poorer educational attainment in adulthood [25].

Considering a more nuanced association bewteen PEMCS and cognition, it has been suggested that PEMCS is more specifically associated with “hot” cognitive tasks that involve stress or frustration [26, 27]. This would be consistent with findings linking this exposure to childhood externalising behavior [28, 29]. Huijbregts et al. found that children with PEMCS showed poorer inhibitory control in a more frustrating task (but not in a regular task), while also showing higher conduct problems and hyperactivity-inattention scores [27]. Difficulties with self-regulation appear to be more pronounced in boys than in girls [30].

Despite the number of studies examining PEMCS and cognition, to our knowledge only two have investigated the association in adulthood [12, 15]. Among 18-year old male Swedish conscripts, Lundberg et al. found that intellectual impairment was associated with PEMCS, though to a reduced degree after controlling for parental factors [15]. A study by Mortensen et al. found that smoking during pregnancy (measured during the third trimester) was associated with lower IQ score in 18-year old males in a dose-response manner, while controlling for parental social status and education and other factors [12].

Clifford et al. have suggested that stronger findings in male-only samples may indicate that any association between PEMCS and cognition in adulthood could be sex-specific with vulnerability in males possibly masked in other analyses by inclusion of a high number of females [24]. Two among the more recent studies showed a significant association between PEMCS and intelligence in males only [12, 14]. There are biological and developmental reasons to suspect sex-specific effects on cognition. Rodent studies have noted differences in neurodevelopment in response to prenatal nicotine according to sex [31, 32]. Studies examining PEMCS in humans have also noted more marked imaging differences in females [33, 34] and behavioral findings that differed between males and females [34–37]. This has included differences in gene by exposure interaction in one study [38]. In addition to these imaging and laboratory findings, males are more at risk for inattention and hyperactivity and other externalizing behaviors that are associated with poorer cognitive outcomes [39].

The relationship between PEMCS and cognition is complex and the potential role of confounders, mediators and moderators of the relationship needs to be considered. Heritable traits such as parental cognitive ability and parental psychiatric vulnerability may particularly confound associations between PEMCS and cognition [24], while maternal use of alcohol in pregnancy may co-occur with PEMCS [11, 19]. Other factors, for example birth weight and family functioning [21], have been suggested as potential mediators or moderators of observed effects.

To date, there has been limited exploration of how adolescent mental health influences the relationship between PEMCS and adult cognition. A range of adolescent psychiatric and behavioral problems has been associated with both PEMCS and with poorer cognitive outcomes, though not in all studies. PEMCS has been associated with adolescent psychotic-like experiences (PLEs) [4] and with schizophrenia [40], though not in all studies [41]. Similarly, PEMCS has been associated with inattention and hyperactivity and other externalizing behaviors [5, 6], though not in all studies [42]. These psychiatric and behavioral problems are also associated with problems in cognition. Young people with PLEs have shown deficits across multiple domains [43]. In addition to poorer performance on tasks requiring attention [44], adults with attention-deficit hyperactivity disorder (ADHD) perform more poorly on tests of general intellectual ability [45]. These findings raise the possibility that PEMCS may affect later cognition through an effect on adolescent emotional and behavioral problems. These problems, including the presence and degree of PLEs, adolescent inattention and hyperactivity and other externalizing behaviors in adolescence, could potentially affect the longitudinal course of cognitive development into early adulthood.

### Aims and objectives

Using a cohort sub-group with groups selected based on PEMCS and matched by maternal education and geographic region, this study aims to clarify the long-term associations between PEMCS and cognition and whether any of these associations are sex-specific. The primary hypothesis is that PEMCS is associated with adverse cognitive outcomes in adulthood but that males are more vulnerable to these adverse outcomes. Where there was evidence in support of the primary hypothesis, we tested three secondary hypotheses: (1) observed associations are not explained by maternal alcohol use during pregnancy, birth weight or current smoking status; (2) observed associations are partly mediated by adolescent mental health status, specifically PLEs, inattention and hyperactivity, and other externalizing behaviors; (3) PEMCS interacts with adolescent mental health status in predicting these cognitive associations.

## Methods

### Study design

The study is a matched subsample selected from a general population-based birth cohort and followed up from pregnancy to early adulthood (aged 25–27 years).

### Study setting

The sample consists of participants recruited from the general population-based Northern Finland 1986 Birth Cohort (NFBC 1986), which included 99% of those born between July 1985 and June 1986 (9,432 live-born children) in the two northernmost regions of Finland (Oulu and Lapland) [46].

### Participants

Of the original sample, 6,985 (74%) participated in the 16-year follow-up. Among this group, there were 698 individuals with PEMCS and eligible for inclusion after consideration of inclusion and exclusion criteria. Inclusion criteria were birth in the north of Finland between 1 July 1985 and 30 June 1986 and both parents living in 2005 (aiming to ultimately examine parents in addition to offspring). Exclusion criteria were adoption, maternal use of alcohol in excess of 4 drinks per week during pregnancy, maternal diabetes during pregnancy, premature birth before 35 weeks, multiple births, post-natal hyperbilirubinaemia requiring transfusion, serious childhood medical illnesses, neurological conditions, developmental conditions, diagnosis of psychosis and intellectual disability (IQ < 70). Full inclusion and exclusion criteria are listed in Lotfipour et al [47]. The non-exposed control group was selected randomly from offspring of non-smoking mothers with the same inclusion and exclusion criteria. Non-exposed controls were matched to the exposed participants by place of birth (urban/rural and Oulu region or Lapland region) and maternal education, using 5-level categories based on combination of basic and occupational education, at time of pregnancy [47].

Of the invited 1,396 eligible participants (698 exposed and 698 matched non-exposed), a total of 471 (34%) completed the full protocol (including magnetic resonance imaging). Among the invited sample, the participation rate was slightly higher among non-exposed (253/698 or 36%) than exposed (218/698 or 31%) participants (Fisher’s exact test: *P* < 0.05), and among female (39%) than male (28%) participants (*P* < 0.001). There was no difference in participation rate according to place of birth or maternal education level.

The Ethics committee of the Northern Ostrobothnia Hospital District in Finland has approved the study. All participants gave written informed consent. The follow up field study with cognitive assessments was conducted during the years 2011–13 when the birth cohort members were young adults (aged 25.4–27.8 with mean age 26.5 years).

### Assessments

#### Cognitive assessments

When participants reached young adulthood (aged 25–27 years), we assessed verbal and non-verbal skills, learning and memory, working memory, attention, decision-making and fine motor skills (see Table 1). Cognitive testing was planned to take 45 min but took up to 75 min in some cases. Verbal skills were tested using the vocabulary component of the Wechsler Adult Intelligence Scale (WAIS-3), while non-verbal skills were tested using the Matrix Reasoning section of the WAIS-3 [48]. Learning and memory were assessed using the Paired Associates Learning (PAL) test from the CANTAB battery [49]. Executive functioning/cognitive flexibility was measuring using Semantic Fluency [50]. Fine motor-skills were measured with time taken to complete the Grooved Pegboard test using dominant hand [51]. Processing speed was measured with time taken to complete the Stroop test [52]. Response inhibition (impulse control) was tested using the modified Stop Signal Test (MSST) from the CANTAB battery, performed on a 5^th^ generation iPod Touch (Apple, Cupertino CA, USA) device. This test involved the participant reacting as quickly as possible to the actions of a screen character with stop signals and measures of various response parameters, including errors, successful stops and stop signal reaction time (SSRT)) (Lumsden J: Comparison of data generated by gamified and standard stop signal tasks, unpublished). The outcome we utilized was the SSRT, after excluding outliers with SSRT < 0.3 s.

**Table 1:** Cognitive domains of interest, tests performed and outcome of interest

Cognitive domain	Cognitive test	Outcome measure
Verbal skills	Vocabulary component of WAIS-3	Z-transformed score
Non-verbal skills	Matrix reasoning component of WAIS-3	Z-transformed score
Learning and memory	Paired Associates Learning from CANTAB battery	Z-transformed inverse of total errors (adjusted)
Executive functioning/cognitive flexibility	Semantic Fluency	Z-transformed number of words named
Fine motor skills	Grooved Pegboard test with dominant hand	Z-transformed inverse of time taken to complete
Processing speed	Stroop test	Z-transformed inverse of time taken to complete
Response inhibition/impulse control	Modified Stop Signal Test from CANTAB battery	Z-transformed inverse of stop signal reaction time

*WAIS-3* Wechsler Adult Intelligence Scale, version 3

*CANTAB* Cambridge Neuropsychological Test Automated Battery

Raw scores of the above tests that were normally distributed (all except for PAL) were transformed to Z-scores with higher scores indicating better performance in each case. Prior to conversion to Z-scores, non-normally distributed variables were transformed. The PAL test was transformed using a square root transformation.

#### Prenatal exposure to maternal cigarette smoking (PEMCS)

PEMCS status was determined prospectively: during antenatal clinic attendance, mothers were asked about their smoking behavior. For the purpose of this study, we defined smoking status during pregnancy as history of maternal smoking in the beginning of pregnancy and continuing exposure to one or more cigarettes per day after the second month of pregnancy (Question used “Did mother smoke after second month of pregnancy?”). The non-exposed group comprised offspring of mothers who had never smoked. In order to examine a possible dose-response relationship between cognitive performance and PEMCS, we also defined smoking according to the number of cigarettes per day (“How many cigarettes?): 1-9 cigarettes or 10 or more cigarettes.

#### Prenatal exposure to alcohol, birth weight and current smoking

Prenatal exposure to alcohol was also determined prospectively during antenatal attendance by asking the mother: “Have you had alcoholic drinks during this pregnancy?” Of note, those who drank in excess of 4 units per week were excluded. Birth weight was recorded at birth and measured in kilograms. Current smoking status of the cohort members was determined at the same time as cognitive testing and converted into a binary variable: any current smoking vs. no current smoking.

#### Mental health status variables

Three variables about mental health status of the adolescent were regarded as potential mediators of any association between PEMCS and cognitive problems in adulthood: psychotic-like experiences (PLEs), inattention and hyperactivity; and other externalizing behaviors. Self-reported PLEs over the previous 6-month period were ascertained using the PROD-screen administered at 16 years of age [53]. Consistent with previous practice [53], we used a conservative cutoff of 3 or more specific items on the PROD-screen to create a binary variable for PLEs. Inattention and hyperactivity were measured using scores derived from the inattention and hyperactivity sections of the SWAN rating scale administered at 16 years of age [54] with results Z-transformed. SWAN measures problems in attention and hyperactivity-impulsivity lasting for at least one month. Other externalizing behaviors over the previous 6 months were assessed using results from the other externalizing scales (aggressive behavior, rule-breaking behavior and intrusive behavior) of the Youth Self Report [55], which were added together, log-transformed for normal distribution and Z-transformed.

### Statistics

We first compared participants with PEMCS with those not exposed in terms of their demographic characteristics (sex, history of prenatal alcohol exposure, current smoking status, birth weight), mental health status in adolescence (PLEs, inattention and hyperactivity Z-score, and other externalizing behaviors Z-score) and basic cognitive performance in adulthood, using chi-squared tests or independent t-tests as appropriate. While there were minimal differences in terms of sex, those with PEMCS were also more likely to be exposed to alcohol at this time (Chi-square = 26.129, *P* < 0.001), they were lighter at birth (T = 3.404, *P* < 0.001) and they were more likely to smoke in young adulthood (Chi-square = 8.275, *P* = 0.004). Overall and across the range of cognitive tests, there was little evidence of differences in cognitive scores, though there was a trend towards poorer performance in terms of vocabulary (T = 1.842, *P* = 0.066) in the exposed vs. non-exposed participants. In addition, those with PEMCS showed poorer mental health status in adolescence (see Table 2 for full details).

**Table 2:** Prenatal exposure to maternal cigarette smoking (PEMCS) and demographic characteristics, cognitive score and potential mediating factors

	No PEMCS	PEMCS	T/Chi-square/Z	*P*-value
Demographic factors				
Male sex	98/253 (39%)	95/218 (44%)	1.136	0.287
Smoking at 26-27 years	80/253 (32%)	97/218 (45%)	**8.275**	**0.004**
Prenatal exposure to alcohol	19/249 (8%)	54/217 (25%)	**26.129**	**<0.001**
Birth weight (kg)	3.643	3.497	**3.404**	**<0.001**
Cognitive z-scores				
Vocabulary	0.079	-0.091	1.842	0.066
Matrix reasoning	0.054	-0.063	1.273	0.204
Verbal fluency	0.027	-0.031	0.628	0.530
Pegboard	0.036	-0.042	0.846	0.398
Stroop	0.011	-0.013	0.251	0.802
PAL	0.039	-0.052	0.979	0.328
MSST	-0.049	0.052	1.036	0.301
Adolescent mental health status				
Inattention and hyperactivity (SWAN) z-score	0.170	-0.203	**3.720**	**<0.001**
PLEs	69/229 (30%)	74/191 (39%)	3.440	0.064
Externalizing (YSR) z-score	-0.185	0.208	**4.072**	**<0.001**

*PAL* Paired Associates Learning, *MSST* Modified Stop Signal Test, *SWAN* Strengths and Weaknesses of ADHD symptoms and Normal behavior, *PLEs* Psychotic-like Experiences, *YSR* Youth Self-Report

Bold = *P* < 0.05

We next tested whether PEMCS was associated with adverse cognitive outcomes in adulthood that differed by sex. This was done using hierarchical multiple regression with normalized performance on cognitive tests as the dependent variable (vocabulary, matrix reasoning, verbal fluency, pegboard test, Stroop test, PAL and MSST). These tested our primary hypothesis and secondary hypotheses 1 and 2. For these tests, we excluded individuals for whom there was incomplete data on a variable of interest, resulting in *n* = 185 for females and *n* = 132 for males. Step 1 used univariate linear regression to examine the sex-specific associations between PEMCS and cognitive outcomes. Two associations were found: PEMCS with vocabulary and matrix reasoning in males. These associations were carried forward to step 2. There were no associations between PEMCS and cognitive performance in females. At this point, we tested observed associations for an interaction between sex and PEMCS using Chi-square test for interaction and performed a non-parametric test for trend (command = nptrend) to assess if there was a dose-response relationship between PEMCS and the cognitive outcome. We then progressed to step 2 with the observed associations in step 1. Step 2 repeated the step 1 analyses but controlled for birth weight, prenatal exposure to alcohol and current smoking status [56]. Step 3 built on step 2 by also including the individual variables for mental health in adolescence. A priori power calculations suggested 36% power in the male group (*n* = 132) and 48% power in the female group (*n* = 275) to detect a small effect size (f^2^ = 0.02) at α = 0.05 [57]. However, in both groups there was >99% power to detect a medium effect size (f^2^ = 0.15) at α = 0.05.

Where associations were observed between PEMCS and cognitive tests, we utilized the results of the hierarchical multiple regression to determine if there was mediation by mental health status. Prior to interpreting these results in our mediation analyses, we examined if basic requirements were met for mediation [58]: (a) confirm that PEMCS predicted adverse cognitive outcomes using regression; (b) confirm that PEMCS predicted the potential mediating variables using regression (steps 1 and 2 of the hierarchical multiple regression); (c) confirm that inclusion of the mediating variable in a regression model with PEMCS and cognition reduces or eliminates the previous effect (step 3 of the hierarchical multiple regression).

Requirement (a) was met in the case of vocabulary and matrix reasoning in males with poorer performance in those with PEMCS (*P* = 0.011 for vocabulary and *P* = 0.026 for matrix reasoning). Requirement (b) was broadly met in the case of all three mental health factors. Controlling for sex, those with PEMCS showed a trend towards higher odds of PLEs in adolescence (*P* = 0.089), and strong evidence for more inattention and hyperactivity in adolescence (*P* < 0.001) and more other externalizing behaviors (*P* < 0.001). We therefore proceeded to requirement (c), a comparison of the results of steps 1 and 2 with those of step 3 in the hierarchical multiple regression. Finally, where a change in association was noted with inclusion, we confirmed mediation statistically using the Sobel test [59].

## Results

### PEMCS and cognitive performance by sex

PEMCS was not associated with adverse cognitive outcomes in adult females and was not associated with adverse cognitive outcomes in four of six tests in adult males (see Table 3). In males, PEMCS was associated with relatively small effects in terms of poorer scores in vocabulary (f^2^ = 0.05, beta coefficient = -0.444, 95% CI: -0.783, -0.104) and matrix reasoning (f^2^ = 0.04, beta coefficient = -0.379, 95% CI: -0.711, -0.047) [60]. Chi-squared tests for interaction, comparing models with and without an interaction term between sex and PEMCS for both vocabulary and matrix reasoning, supported an interaction between the two factors in predicting vocabulary score (Chi square = 6.75, *P* = 0.009) and showed a trend towards interaction in predicting matrix reasoning score (Chi-square = 3.27, *P* = 0.071). There was evidence for a dose-response trend in the case of matrix reasoning (Z = 2.89, *P* = 0.004) but insufficient evidence to support a dose-response trend for vocabulary (Z = 1.60, *P* = 0.109).

**Table 3:** Prenatal exposure to maternal cigarette smoking (PEMCS) and cognitive outcomes in adulthood (vocabulary, matrix reasoning, verbal fluency, pegboard test, Stroop test, PAL and MSST test), according to sex

Cognitive test	Males (*n* = 132)^a^		Females (*n* = 181)^b^	
	Beta Coefficient (95% confidence interval)	*P*-Value	Beta Coefficient (95% confidence interval)	*P*-Value
Vocabulary	**-0.444 (-0.783, -0.104)**	**0.011**	0.123 (-0.150, 0.396)	0.375
Matrix reasoning	**-0.379 (-0.711, -0.047)**	**0.026**	0.026 (-0.265, 0.316)	0.863
Verbal fluency	-0.256 (-0.615, 0.102)	0.160	-0.058 (-0.350, 0.233)	0.692
Pegboard	-0.068 (-0.339, 0.203)	0.621	-0.164 (-0.385, 0.057)	0.145
Stroop	-0.143 (-0.472, 0.186)	0.392	0.083 (-0.199, 0.366)	0.562
PAL	-0.232 (-0.592, 0.128)	0.204	0.014 (-0.296, 0.324)	0.929
MSST	-0.092 (-0.416, 0.234)	0.578	0.023 (-0.319, 0.364)	0.896

*PAL* Paired Associates Learning, *MSST* Modified Stop Signal Test

^a^ Except for MSST where n = 118

^b^ Except for MSST, where *n* = 166

Bold = *P* < 0.05

#### Mediation analysis

As indicated in Table 4, there was evidence in support of our first secondary hypothesis that observed associations were not mediated by prenatal exposure to alcohol, low birth weight or current smoking status with only marginal changes in the beta coefficients after controlling for these factors.

**Table 4:** Effect of potential mediating factors on the relationship between prenatal exposure to maternal cigarette smoking (PEMCS) and vocabulary and matrix reasoning in males (*n* = 132)

Model	Vocabulary		Matrix reasoning	
	Coefficient (95% confidence interval)	*P*-value	Coefficient (95% confidence interval)	*P*-value
Model 1^a^	**-0.444 (-0.783, -0.104)**	**0.011**	**-0.379 (-0.711, -0.047)**	**0.026**
Model 2^b^	**-0.456 (-0.789, -0.122)**	**0.008**	**-0.380 (-0.715, -0.046)**	**0.026**
Model 2 + PLEs	**-0.460 (-0.794, -0.123)**	**0.007**	**-0.383 (-0.719, -0.048)**	**0.025**
Model 2 + inattention and hyperactivity Z-score	**-0.382 (-0.730, -0.034)**	**0.032**	-0.300 (-0.648, 0.048)	0.091
Model 1 + other externalizing behavior Z-score	**-0.527 (-0.862, -0.192)**	**0.002**	**-0.395 (-0.737, -0.053)**	**0.024**

^a^ Model 1 coefficient is association between PEMCS and vocabulary or matrix reasoning in males

^b^ Model 2 coefficient is association between PEMCS and vocabulary or matrix reasoning in males, controlling for prenatal exposure to maternal use of alcohol in pregnancy, birth weight and current smoking status

Bold = *P* < 0.05

*PLEs* Psychotic-like Experiences

There was mixed evidence regarding secondary hypothesis number two that observed associations are partly mediated by adolescent mental health status in adolescence. There was no evidence that the association between PEMCS and either of the two cognitive scores in males was mediated by PLEs in adolescence. In the case of inattention and hyperactivity, there was a suggestion for potential mediation of the relationship with vocabulary (beta reduced from -0.456 to -0.383) and matrix reasoning (beta reduced from 0.380 to 0.300). However, Sobel test provided no evidence for mediation (vocabulary - 19.8% mediated, *P* = 0.135; matrix reasoning – 22.6% mediated, *P* = 0.137).

#### Interaction analyses

The evidence was mixed regarding the hypothesis that PEMCS interacts with mental health status in adolescence in predicting observed associations. There was no evidence for interaction between PEMCS and any mental health factor (*P* = 0.798 for PLEs, *P* = 0.231 for inattention and hyperactivity, *P* = 0.952 for other externalizing behaviors) in the case of vocabulary scores. There was no evidence for interaction between PEMCS and two of the mental health factors (*P* = 0.919 for inattention and hyperactivity and *P* = 0.810 for other externalizing behaviors) in predicting matrix-reasoning scores. PLEs interacted with PEMCS in predicting matrix-reasoning performance (Chi-square = 3.98, *P* = 0.046). Specifically, PEMCS predicted poorer performance in matrix reasoning among those without PLEs in adolescence (beta = -0.606, 95% CI: -1.001, -0.205) but not among those with PLEs in adolescence (beta = -0.119, 95% CI: -0.624, 0.386).

## Discussion

In this large general population-based and well-controlled sample, PEMCS was not associated with poorer cognitive scores across most cognitive tests performed in males and all cognitive tests performed in females. In adult males, PEMCS was associated with a small effect in terms of poorer performance on vocabulary and matrix reasoning. These associations were partly mediated by adolescent inattention and hyperactivity and showed differences depending on the presence or absence of PLEs in adolescence.

Our finding that there were no significant associations between PEMCS and cognition in females and only limited and small associations in males is consistent with the observation of nuanced associations described elsewhere [24]. Earlier studies have not controlled sufficiently for confounding factors, including socioeconomic status and use of other substances in pregnancy. This study was matched on maternal education and controlled for alcohol use during pregnancy, factors that may have influenced positive findings in other studies. Our finding of no associations in females across all tests, including both traditional pencil and paper tests and computer -based tests, adds to the consistency of the negative findings in females. Furthermore, the coefficients and confidence intervals in these tests were close to zero, supporting the absence of associations in these cases. This is consistent with suggestions that although PEMCS may exert structural and functional effects [34], this may not result in any measurable cognitive effects [19]. We know that PEMCS results in prenatal exposure to a variety of toxic compounds, of which nicotine is but one, along with reduced uterine blood flow with associated episodes of foetal hypoxia and malnutrition [61]. However, significant brain development follows birth with associated multiple opportunities for brain adaptation to early insult, particularly if these insults are mild. This may explain the general lack of associations between PEMCS and cognition in our sample.

Our results regarding the negative effects of PEMCS on aspects of male cognition replicate those of Mortensen et al., who noted that heavy smoking reduced intelligence scores in young adult males by 0.41 standard deviations [12]. Indeed, we have found that PEMCS was associated with poorer performance on aspects of intelligence in adult males: by 0.44 standard deviations in the case of vocabulary scores and by 0.38 standard deviations in the case of matrix reasoning scores. While it is more difficult to compare results to those found in children, Braun et al. also noted a differential effect on intelligence scores in 7-year old males [14]. Our findings in relation to males are further supported by evidence for a dose response trend in the case of matrix reasoning and a trend towards this in the case of vocabulary.

Our findings must be viewed in the context of a growing body of evidence suggesting differences in male and female responses to PEMCS. Though many imaging studies have not noted sex differences in response to PEMCS [62], studies of particular regions have noted more pronounced PEMCS-related differences in female than male offspring with reduced corpus callosum size [33] and cortical thinning in several regions [34]. The latter finding was also associated with differences in self-rated assessment of caring in females but not males. At a physiological level, rodent studies have found that prenatal exposure to nicotine was associated with reduced number of serotonin transporter binding sites in the cerebral cortex that is more marked in females than males [32]. On the other hand, males but not females exposed to prenatal nicotine showed increased number of 5HT1A receptors in their cerebral cortex in another study [31]. Studies of behavioral outcomes have also noted sex differences, with prenatal exposure to nicotine reducing locomotor activity in male but not female rodents [63]. In human adolescents, PEMCS has been associated with more pronounced conduct problems in males than females [35, 36]. Indeed, genetic studies have noted a sex-specific pattern of gene-by-exposure interaction in predicting conduct disorder symptoms [38]. Our findings therefore add to existing evidence of differences in response to PEMCS by sex. Stronger associations in males between PEMCS and language and intelligence skills (adulthood) and conduct problems (adolescence) may be one manifestation of a more fundamental neural process that has yet to be identified. One potential mechanism is reward sensitivity and emotion regulation, which have been shown to be more vulnerable to the effects of PEMCS in boys than in girls [30].

The partial mediation of our findings by inattention and hyperactivity has a number of possible explanations. The deficits in reward sensitivity and emotion regulation discussed above [30] may play a role. On a related vein, poorer learning could arise from difficulties in performance of “hot” executive function tasks during childhood, difficulties that have been associated with PEMCS and externalizing behaviors [64]. A further possibility is that inattention and hyperactivity is a proxy for another factor in its mediation. A recent sibling study found that PEMCS was not a strong causal factor for ADHD in childhood and adolescence within families, suggesting previous findings may be due to uncontrolled shared genetics or family environment [42]. The role of family environment as a potential mediating factor in cognitive outcomes has also been suggested [21]. Certainly difficulties with inattention and hyperactivity could affect educational achievement, reflected in testing for vocabulary and matrix reasoning. It remains to be seen if addressing childhood inattention and hyperactivity could prevent longer-term cognitive effects of PEMCS.

Previous studies have highlighted the value of identifying interactions in understanding how PEMCS may influence cognitive performance [21] in order to identify who might benefit from intervention. We have identified interactions between PEMCS and both sex and adolescent PLEs that may prove useful at a public health level. The finding in relation to PLEs is somewhat counter-intuitive. The lack of an association between PEMCS and cognition in those with PLEs may reflect the exclusion of those later treated for serious mental illness from this study [47]. Those with PLEs excluded from this study were likely to have poorer long-term outcomes and significant further risk factors that may affect cognition. The remaining individuals with PLEs included in this study may therefore be a particularly resilient group. The findings may also be due to chance, though the Chi-square test for interaction is a particularly conservative test.

Our study has a number of features that add weight to the findings above. Firstly, the sample was matched to reduce the possible role of parental socio-economic status or maternal education. Secondly, the sample has been followed prospectively from birth and we have been able to consider mental health status in adolescence, almost 10 years prior to cognitive testing. This allowed for the consideration of adolescent mental health in its temporal context, unaffected by recall bias. Thirdly, the sample includes both males and females, allowing for separate analysis of these groups to confirm or refute previous theories on susceptibility to neurodevelopmental insult.

Limitations of this study include that the size of our sample provided insufficient power to detect small effects on cognitive domains in males and females. However, our findings are consistent with previous studies. Study of smaller effects requires study in a sample of 550 per group [57]. While the exclusion criteria in this study address many potential confounding factors, they also reduce the generalizability of the findings. Exclusion criteria meant that the sample were relatively healthy in terms of mental health and cognitive performance. For example, we cannot comment on the role of PEMCS on intellectual disability as individuals with an IQ < 70 were excluded and we cannot fully consider the role of mental health given the exclusion of those with more serious mental disorders. This is related to a further limitation, which is that over half of those invited did not participate. Those not completing may differ from those who completed. For example, we know that more without PEMCS than with PEMCS participated. This may contribute to a healthy participant bias, with findings not reflecting the experience of those with worse outcomes and results being potentially more conservative than would be the case at population level. A further limitation is that our measure of PEMCS is based on self-report of use rather than objective measures. This could result in inclusion of exposed participants who denied use in the non-exposed group with further potential to dilute any observed findings. Finally, though primary analyses were hypothesis driven, the number of tests performed in post-hoc analyses examining mediation and interaction mean that chance is a possible explanation for some of the findings. Indeed, most primary findings were negative and positive findings must be regarded as tentative and in need of replication in larger samples and different settings.

The findings of this study have several public health and research implications. Firstly, they add further weight to public health efforts to eliminate smoking in pregnancy as they suggest long-term adverse consequences of this behavior. From a research perspective, the results of this study add to findings suggesting the need for more nuanced analysis of the long-term effects of smoking, particularly in the area of cognition. Further analysis of potential mediating factors during development that may affect adult cognition, such as reward sensitivity and emotional regulation [30] or difficulties with “hot” cognition tasks [27], could provide targets to improve adult outcomes. Finally, these results highlight the need for further study on factors affecting cognition in those at risk for psychosis, including replication of these methods in a sample including individuals who progressed to develop significant mental illness in order to identify preventable causes of poorer functional outcomes in this population.

## Conclusions

In conclusion, PEMCS was not associated with medium to strong effects on cognitive scores across most cognitive tests performed in males and all cognitive tests performed in females. In adult males, PEMCS was associated with a small effect in terms of poorer performance on vocabulary and matrix reasoning. Post-hoc analysis of these small effects tentatively suggested that the associations were partly mediated by adolescent inattention and hyperactivity. Furthermore, this association in males differed according to adolescent PLEs, though this may be related to the sampling methodology. These findings add weight to public health measures to eliminate smoking in pregnancy, while also suggesting that in terms of cognition, adults, especially females, are perhaps surprisingly flexible in adapting to exposure to smoking in pregnancy.
